# Phenol- and resorcinol-appended metallocorroles and their derivatization with fluorous tags

**DOI:** 10.1038/s41598-022-23889-0

**Published:** 2022-11-10

**Authors:** Abraham B. Alemayehu, Abhik Ghosh

**Affiliations:** Department of Chemistry, University of Tromsø, N-9037 Tromsø, Norway

**Keywords:** Chemistry, Materials science

## Abstract

Boron tribromide-mediated demethylation of rhenium-oxo and gold *meso*-tris(4-methoxyphenyl)corrole and *meso*-tris(3,5-dimethoxyphenylcorrole), M[T*p*OMePC] and M[T(3,5-OMe)PC] (M = ReO, Au), have yielded the corresponding phenol- and resorcinol-appended metallocorroles, M[T*p*OHPC] and M[T(3,5-OH)PC]**,** in good yields. The latter compounds proved insoluble in dichloromethane and chloroform but soluble in THF. The M[T(3,5-OH)PC] derivatives also proved moderately soluble in 0.05 M aqueous KOH. Unlike oxidation-prone aminophenyl-substituted corroles, the phenol- and resorcinol-appended metallocorroles could be readily handled in air without special precautions. The phenolic metallocorroles could be readily alkylated with 4,4,5,5,6,6,7,7,8,8,9,9,10,10,11,11,11-heptadecafluoroundecyl iodide (“FtI”) to afford the fluorous-tagged metallocorroles M[T*p*OFtPC] and M[T(3,5-OFt)PC] in > 90% yields. The simplicity of the synthetic protocols promise a wide range of phenolic and fluorous-tagged porphyrin analogues with potential applications to diverse fields such as sensors, catalysis, and photodynamic therapy, among others.

## Introduction

**Figure 1:**
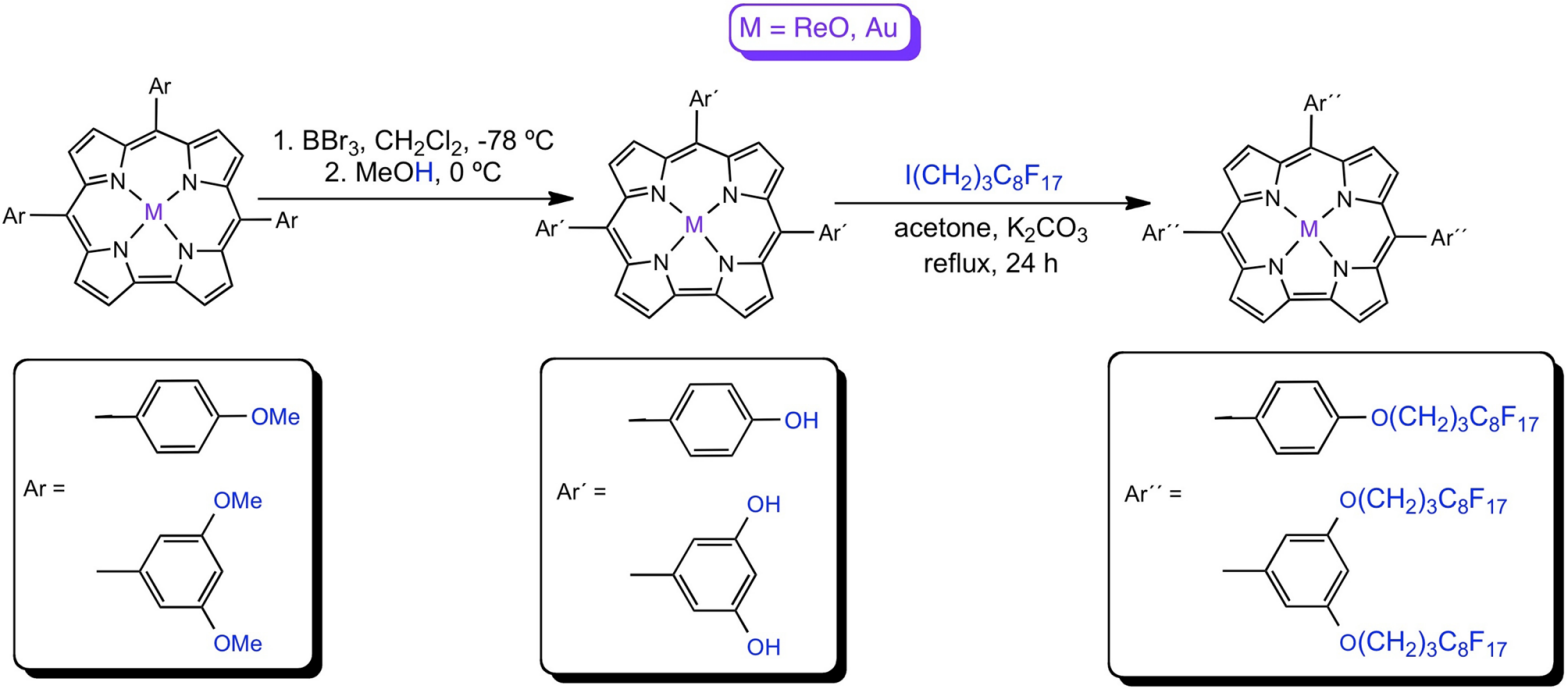
Schematic illustration of the synthesis of phenol- and resorcinol-appended metallocorroles and their derivatization with a fluorous tag.

Corroles, which were mere curiosities just 25 years ago^1–5^, are now a major class of macrocyclic ligands with applications rivaling those of porphyrins^6,7^. Besides key photophysical properties^8–15^, biomedical applications such as photodynamic therapy (PDT)^16–19^ require water-soluble and amphiphilic ligands for effective biodelivery^20–28^. An attractive approach to effective biodelivery in PDT involves nanodroplets of locally-perfluorinated (fluorous^29–35^) porphyrin analogues dissolved in a fluorocarbon solvent with high oxygen-carrying capacity. Finally, new strategies for functionalization are a key first step for novel bio- and nanoconjugation of porphyrin analogues^36–42^. Against this backdrop, we present here simple synthetic routes to amphiphilic phenol- and resorcinol-appended metallocorroles^43^ (analogous to other similarly functionalized porphyrin analogues^44–47^) and their elaboration to highly fluorophilic fluorous-tagged derivatives (Fig. 1).

## Results and discussion

### Synthesis of phenol- and resorcinol-appended metallocorroles

**Figure 2:**
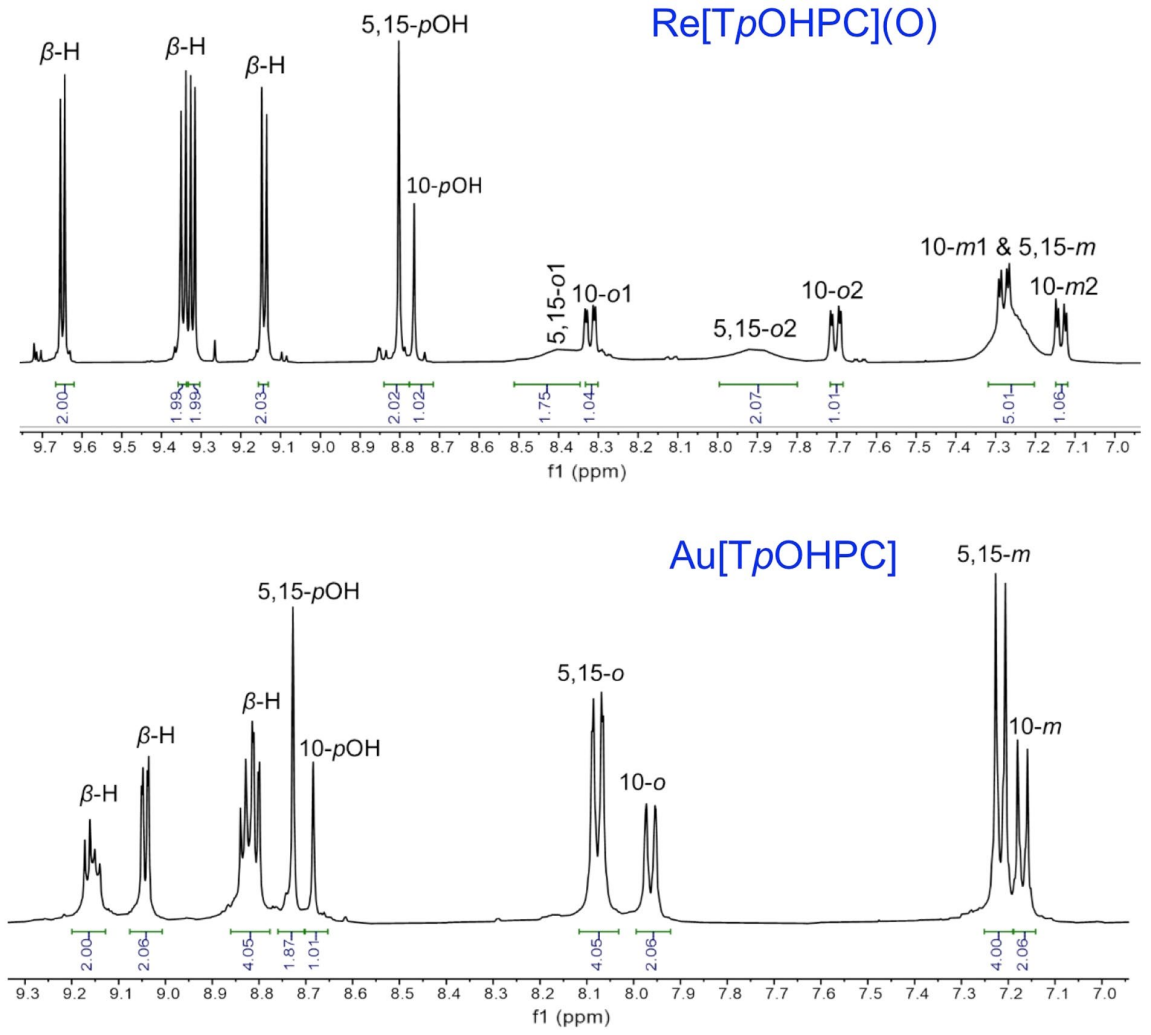
^1^H NMR spectra of M[T*p*OHPC] in THF-*d*_*8*_: M = ReO (above) and Au (below).

**Figure 3:**
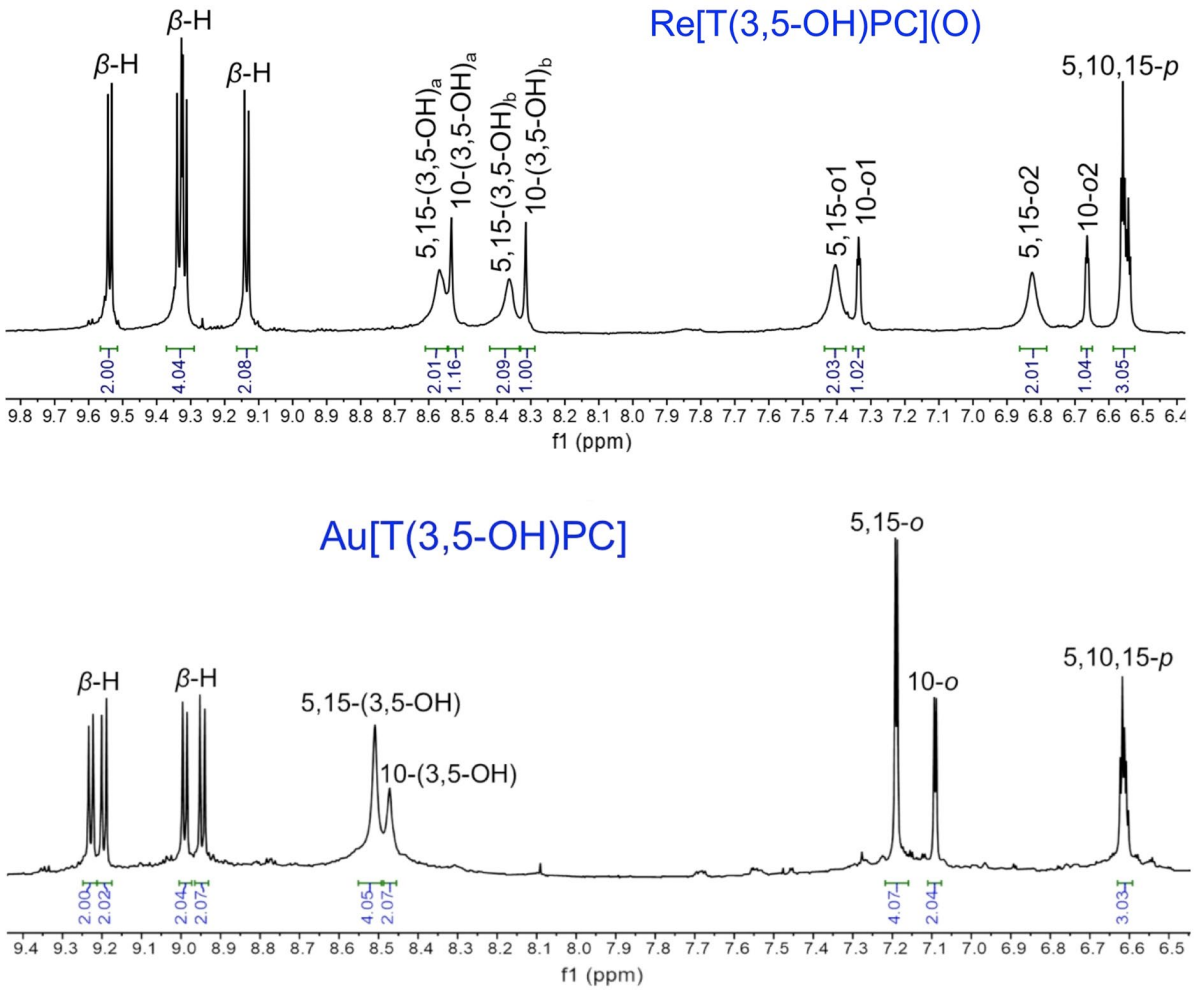
^1^H NMR spectra of M[T(3,5-OH)PC] in THF-*d*_*8*_: M = ReO (above) and Au (below).

Rhenium-oxo^48–53^ and gold^54–63^ tris(4-methoxyphenyl)corrole, M[T*p*OMePC] (M = ReO, Au), and tris(3,5-dimethoxyphenyl)corrole, M[T(3,5-OMe)PC] (M = ReO, Au), which rank among the most readily accessible 5d metallocorroles^64–69^, were used as starting materials. The choice of the two metals was dictated by the fact that they yield rugged, electronically innocent complexes that have been shown to act as triplet photosensitizers in oxygen sensing and in vitro photodynamic therapy experiments. The complexes underwent smooth demethylation^70–73^ with boron tribromide in dichloromethane at – 78 °C, affording phenol- and resorcinol-appended metallocorroles M[T*p*OHPC] and M[T(3,5-OH)PC] in 55 to > 90% yields, with the higher yields observed for M = ReO. The products were purified via silica-gel column chromatography, followed by recrystallization, and characterized by UV–vis spectroscopy, ^1^H NMR spectroscopy, and high-resolution electrospray ionization mass spectrometry. ^1^H NMR spectra of the new compounds indicated complete disappearance of the methoxy protons at around 4 ppm and the appearance of two new singlets between 8.46 and 8.80 ascribable to hydroxy protons (Figs. 2, 3). HRMS proved consistent with the expected structural assignments and also indicated the absence of partially demethylated products and also of higher-mass byproducts.

**Figure 4:**
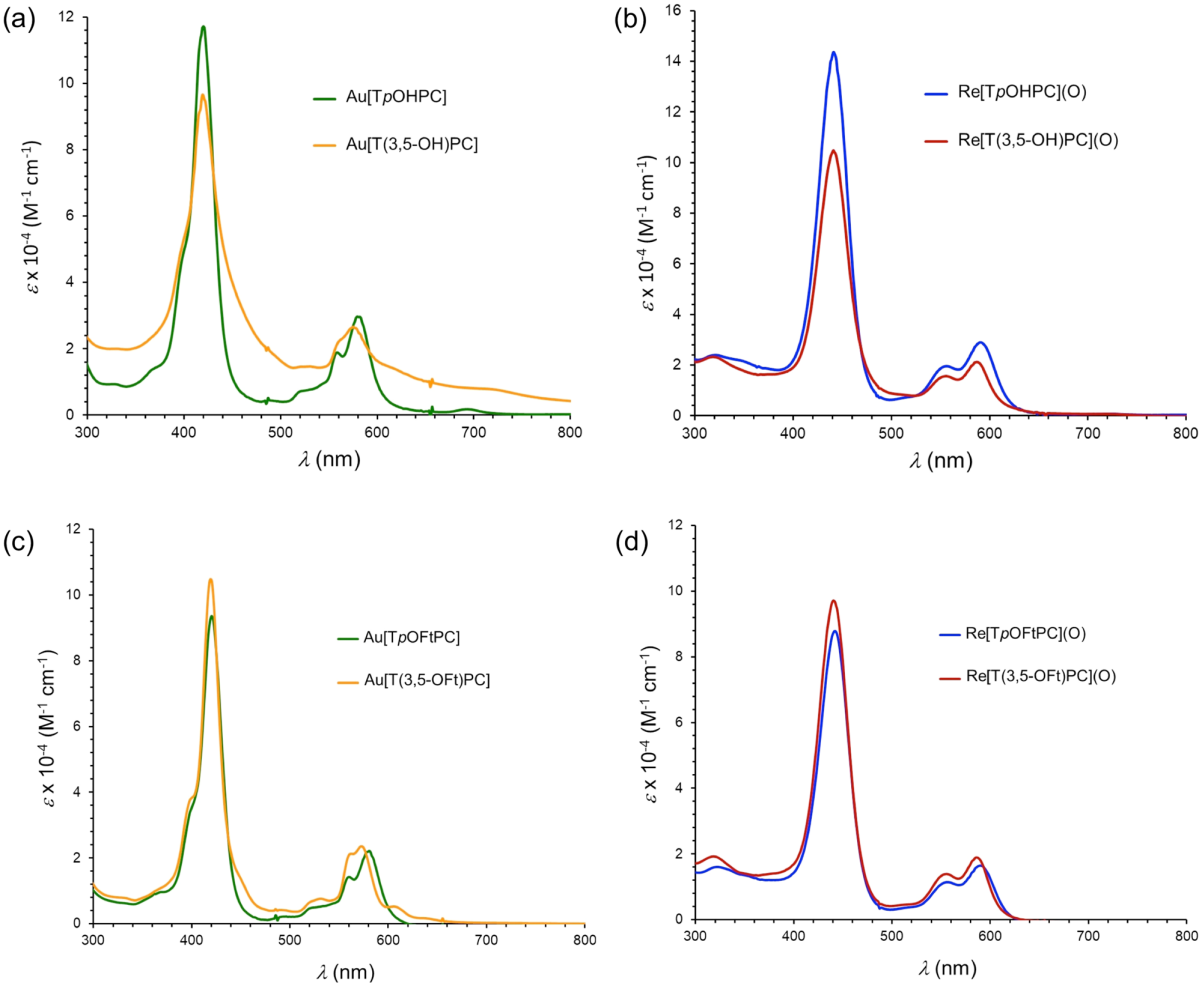
UV–vis spectra of new compounds prepared in this study. Phenolic and fluorous-tagged metallocorroles were analyzed in THF and dichloromethane (with a drop of hexafluorobenzene), respectively. Sample concentrations were in the 10–20 μM range.

The phenolic metallocorroles proved insoluble in dichloromethane and chloroform so UV–vis (Table 1 and Fig. 4) and ^1^H NMR spectra were acquired in THF and THF-*d*_*8*_, respectively. As far as UV–vis spectra are concerned, the phenolic metallocorroles were found to exhibit very similar peak positions relative to their methoxy precursors. In contrast, modest differences in peak positions were observed between the 4-methoxy/hydroxy and 3,5-dimethoxy/dihydroxy derivatives. Interestingly, the resorcinol-appended complexes M[T(3,5-OH)PC] (M = ReO, Au) were found to exhibit moderate solubility and modest spectral redshifts in 0.05 M aqueous KOH, consistent with (partial) deprotonation of the phenolic OH groups^74–78^.

**Table 1:** UV–vis absorption maxima (*λ*, nm) and extinction cofficients [*ε* × 10^–4^ (M^−1^ cm^−1^)].

Complex	Solvent	B	Q
Re[T*p*OMePC](O)	CH_2_Cl_2_	441 (10.84)	556 (1.79) 592 (2.29)
Re[T*p*OHPC](O)	THF	441 (14.35)	556 (1.95), 591 (2.98)
Re[T*p*OHPC](O)^a^	KOH/H_2_O	452	556, 607
Re[T*p*OFtPC](O)^b^	CH_2_Cl_2_/C_6_F_6_	442 (8.78)	556 (1.14), 590 (1.64)
Au[T*p*OMePC]	CH_2_Cl_2_	420 (8.34)	560 (1.76), 580 (1.92)
Au[T*p*OHPC]	THF	420 (11.70)	559 (1.88), 580 (2.95)
Au[T*p*OHPC]^a^	KOH/H_2_O	–	–
Au[T*p*OFtPC]^b^	CH_2_Cl_2_/C_6_F_6_	420 (9.35)	560 (1.42), 580 (2.20)
Re[T(3,5-OMe)PC](O)	CH_2_Cl_2_	441 (9.82)	553 (1.65), 585 (2.24)
Re[T(3,5-OH)PC](O)	THF	440 (10.96)	556 (1.65), 587 (2.24)
Re[T(3,5-OH)PC](O)	KOH/H_2_O	447 (2.68)	565 (0.59), 591 (0.75)
Re[T(3,5-OFt)PC](O)^b^	CH_2_Cl_2_/C_6_F_6_	440 (9.17)	555 (1.39), 585 (1.89)
Au[T(3,5-OMe)PC]	CH_2_Cl_2_	418 (12.81)	561 2.42), 572 (2.63)
Au[T(3,5-OH)PC]	THF	419 (9.65)	576 (2.64)
Au[T(3,5-OH)PC]	KOH/H_2_O	423 (5.08)	581 (1.68)
Au[T(3,5-OFt)PC]^c^	CH_2_Cl_2_/C_6_F_6_	419 (10.48)	562 (2.13), 572 (2.35)

^a^Poor solubility did not allow a determination of spectral data, in part or in whole.

^b^Spectra were acquired in CH_2_Cl_2_ containing a small amount of hexafluorobenzene (C_6_F_6_).

### Synthesis of fluorous-tagged metallocorroles

**Figure 5:**
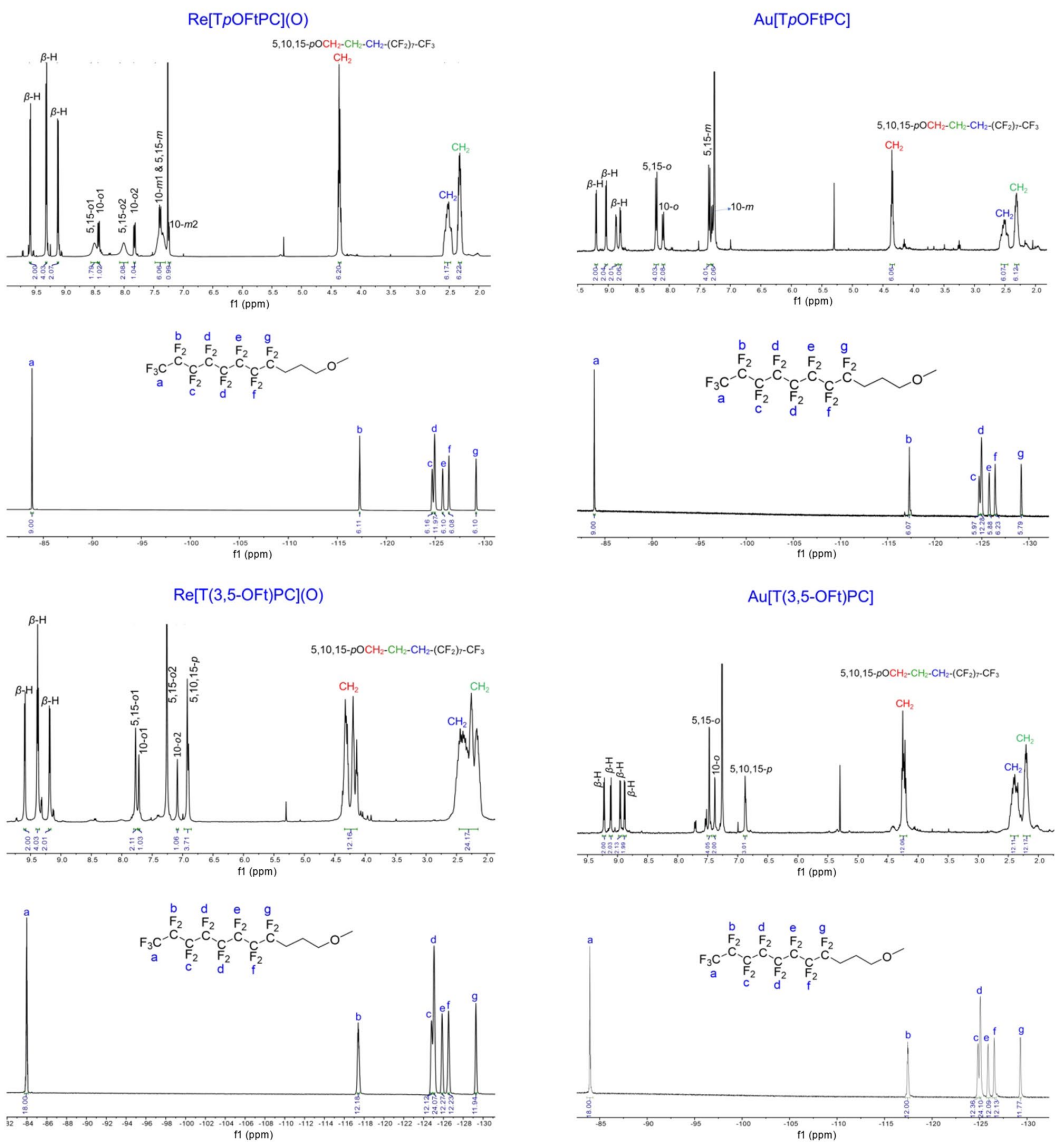
^1^H and ^19^F NMR spectra of M[T*p*OFtPC] and M[T(3,5-OFt)PC] (M = ReO, Au).

The phenol- and resorcinol-appended metallocorroles could be readily derivatized with the fluorous-tagging reagent 4,4,5,5,6,6,7,7,8,8,9,9,10,10,11,11,11-heptadecafluoroundecyl iodide (“FtI”) and K_2_CO_3_ in refluxing acetone over 24 h, whereupon the fluorous-tagged products M[T*p*OFtPC] and M[T(3,5-OFt)PC] (M = ReO, Au) were obtained in > 90% yields. Upon removal of the solvent from the reaction mixture, the residues were dissolved in a small quantity of hexafluorobenzene and purified via column chromatography on a silica gel column with dichloromethane as eluent (i.e., the mobile phase was effectively dichloromethane with a small quantity of hexafluorobenzene). The products were found to be freely soluble in hexafluorobenzene but sparingly so in nonfluorinated solvents including dichloromethane and chloroform. Evidence for exhaustive fluorous tagging came from both ^1^H and ^19^F NMR spectroscopy (in CDCl_3_ with a drop of hexafluorobenzene) and HRMS (Fig. 5). ^1^H NMR spectral analyses showed the complete disappearance of the OH singlets between 8.46 and 8.80 ppm and the appearance of new alkyl proton signals between 2.08 and 4.39 ppm and with an intensity (relative to corrole protons) that exactly matched the expected structure. Clean ^19^F NMR spectra further confirmed this conclusion. Electrospray ionization HRMS also did not reveal any evidence of incompletely fluorous-tagged products. For UV–vis spectroscopy, the fluorous-tagged metallocorroles were dissolved in a minimum volume of hexafluorobenzene followed by dilution with dichloromethane to the required volume. The spectra, unsurprisingly, proved similar to those of simple 5d metallocorroles, with Soret maxima at ~ 420 nm for M = Au and at ~ 440 nm for M = ReO and the usual double-humped Q bands.

## Concluding remarks

In summary, we have described simple routes to amphiphilic phenol- and resorcinol-appended rhenium-oxo and gold corroles, which could be readily derivatized to highly fluorophilic fluorous-tagged derivatives. Although straightforward in retrospect, the successful synthesis of phenolic metallocorroles was *a priori* somewhat uncertain; corroles are more electron-rich than analogous porphyrins and it was unclear whether appending electron-rich phenol and resorcinol moieties would lead to fragile, air-sensitive products. Thus, compared with aminophenyl-substituted porphyrins^79–82^, aminophenyl-substituted corroles are far more susceptible to aerial oxidation and accordingly far trickier to handle^83–86^. These fears proved unfounded for our compounds, which could be readily manipulated in air without special precautions.

The simple access to fluorous-tagged metallocorroles promises a wide range of related products in the near future. These include (a) fluorous-tagged complexes with other metals, including electroactive metals such as manganese and iron^87–89^, (b) light fluorous-tagged complexes with one or two fluorous tags based on so-called A_2_B triarylcorroles^90,91^, and (c) environmentally friendlier (i.e., more biodegradable) complexes with shorter fluorinated chains^92–95^.

The above products and their logical successors promise a plethora of applications, in fields as diverse as sensors^89,96^ photodynamic and related therapies^97,98^, and catalysis^99–101^, among others.

## Experimental section

### Materials

All chemicals were purchased from Sigma Aldrich (Merck). Silica gel 60 (0.04–0.063 mm particle size, 230–400 mesh) was employed for flash chromatography. Metallocorrole starting materials were prepared as previously described^48,56,60^.

#### Re[T(3,5-OCH_3_)PC](O)

This previously unreported complex was prepared from H_3_[T(3,5-OMe)PC], dirhenium decacarbonyl (Re_2_(CO)_10_, 2 equiv), and K_2_CO_3_ (100 mg) using the standard method for Re insertion^48^. Yield 90.12 mg (70.23%). UV–vis (CH_2_Cl_2_) λ_max_ (nm) and ε × 10^–4^ (M^−1^ cm^−1^): 441 (11.40), 554 (1.77), 586(2.28). ^1^H NMR (400 MHz, 25 °C, CDCl_3_): *δ* 9.60 (d, 2H, ^3^*J*_HH_ = 4.4 Hz, *β*-H); 9.41 (d, 2H, ^3^*J*_HH_ = 4.4 Hz, *β*-H); 9.40 (d, 2H, ^3^*J*_HH_ = 4.9 Hz, *β*-H); 9.20 (d, 2H, ^3^*J*_HH_ = 4.9 Hz, *β*-H); 7.77 (s, 2H, 5,15(3,5-*o*1Ph)); 7.71 (s, 1H, 10(3,5-*o*1Ph)_a_); 7.27 (s, 2H, 5,15(3,5-*o*2Ph)); 7.08 (s, 1H, 10(3,5-*o*2Ph)); 6.93 (m, 3H, 5,10,15-*p*Ph); 4.07 (brs, 6H, 5,15(3,5-OCH_3_)_a_; 4.04 (s, 3H, 10(3,5-OCH3)_a_); 3.95 (brs, 6H, 5,15(3,5-OCH3)_b_); 3.90 (s, 3H, 10(3,5-OCH_3_)_b_). HRMS (ESI): [M^−^] = 906.2126 (expt), 906.2133 (calcd for C_37_H_23_N_4_O_7_Re, major isotopomer).

### Instrumental methods

The instrumentation used was essentially the same as in our earlier work^50–53^. UV–visible spectra were recorded on an HP 8453 spectrophotometer. ^1^H NMR spectra were recorded on a 400 MHz Bruker Avance III HD spectrometer equipped with a 5 mm BB/1H SmartProbe in either CDCl_3_ (referenced to residual CHCl_3_ at 7.26 ppm) or THF-*d*_*8*_ (referenced to residual C_4_H_8_O at 3.58 and 1.73 ppm. ^19^F NMR spectra were acquired on the same spectrometer and referenced to hexaflurobenzene (C_6_F_6_, – 164.9 ppm). High-resolution electrospray-ionization mass spectra were recorded on methanolic solutions on an Orbitrap Exploris 120 (Thermo Fisher Scientific) spectrometer.

#### General synthetic procedure for metallotris(4-hydroxyphenyl)corrole complexes, M[T*p*OHPC] (M = ReO, Au)

To a solution of boron tribromide (1.51 mmol) in dry dichloromethane (10 mL) cooled to − 78 °C was added M[T*p*OMePC] (M = ReO, Au; 0.121 mmol), also dissolved in dry dichloromethane (10 mL), over a period of 20 min. The mixture was stirred for 2 h at − 78 °C and then for an additional 12 h at 25 °C. The solution was then cooled to 0 °C in an ice bath and methanol was added to quench any remaining boron tribromide. The reaction mixture was rotary-evaporated to dryness and the solid residue obtained was chromatographed on a silica gel column with 95:5 v/v dichloromethane/methanol as eluent. The final product was recrystallized from 5:1 v/v chloroform/methanol. Yields and spectroscopic data are given below.

#### Synthesis of metallotris(3,5-dihydroxyphenyl)corrole complexes, M[T(3,5-OH)PC] (M = ReO, Au)

To a solution of boron tribromide (3.02 mmol) in dry dichloromethane (10 mL) cooled to − 78 °C was added M[T(3,5-OMe)PC] (M = ReO, Au; 0.121 mmol), also dissolved in dry dichloromethane (10 mL), over a period of 20 min. The mixture was stirred for 2 h at − 78 °C and then for an additional 12 h at 25 °C. The solution was then cooled to 0 °C in an ice bath and methanol was added to quench any remaining boron tribromide. The reaction mixture was rotary-evaporated to dryness and the solid residue obtained was chromatographed on a silica gel column with 9:1 v/v dichloromethane/methanol as eluent. The final product was recrystallized from 3:1 v/v chloroform/methanol. Yields and spectroscopic data are given below.

#### General synthesis of M[T*p*OFtPC] (M = ReO, Au)

A 250-mL round-bottom flask equipped with a stir-bar and a reflux condenser was charged with M[T*p*OHPC] (0.026 mmol), 4,4,5,5,6,6,7,7,8,8,9,9,10,10,11,11,11-heptadecafluoroundecyl iodide (49 mg, 3.2 equiv, 0.083 mmol), and potassium carbonate (100 mg dissolved in 50 mL acetone). The reaction mixture was then refluxed for 24 h, followed by removal of the solvent under reduced pressure. The crude product was dissolved in a minimum amount of hexafluorobenzene and loaded onto a silica gel column and eluted with dichloromethane, affording the desired fluorous-tagged metallocorroles.

#### General synthesis of M[T(3,5-OFt)PC] (M = ReO, Au)

A 250-mL round-bottom flask equipped with a stir-bar and reflux condenser was charged with M[T(3,5-OH)PC] (0.026 mmol), 4,4,5,5,6,6,7,7,8,8,9,9,10,10,11,11,11-heptadecafluoroundecyl iodide (98 mg, 6.4 equiv, 0.167 mmol), and potassium carbonate (200 mg dissolved in 50 mL acetone). The reaction mixture was then refluxed for 24 h, followed by removal of the solvent under reduced pressure. The crude product was dissolved in a minimum amount of hexafluorobenzene and loaded onto a silica gel column and eluted with dichloromethane, affording the desired fluorous-tagged metallocorroles.

#### Re[T*p*OHPC](O)

Yield 88.5 mg (87.2%). UV–vis (THF) λ_max_ (nm) and ε × 10^–4^ (M^−1^ cm^−1^): 441 (14.35), 556(1.95), 591(2.98). ^1^H NMR (400 MHz, 25 °C, THF-*d*_*8*_): *δ* 9.64 (d, 2H, ^3^*J*_HH_ = 4.4 Hz, *β*-H); 9.34 (d, 2H, ^3^*J*_HH_ = 4.8 Hz, *β*-H); 9.32 (d, 2H, ^3^*J*_HH_ = 4.4 Hz, *β*-H); 9.14 (d, 2H, ^3^*J*_HH_ = 4.8 Hz, *β*-H); 8.80 (s, 2H, 5,15-*p*OHPh); 8.76 (s, 1H, 10-*p*OHPh); 8.39 (br s, 2H, 5,15-*o*1Ph); 8.32 (dd, 1H, ^3^*J*_HH_ = 8.0, 2.32 Hz, 10-*o*1Ph); 7.90 (br s, 2H, 5,15-*o*2Ph); 7.74 (dd, 1H, ^3^*J*_HH_ = 8.2, 2.32 Hz, 10-*o*2Ph); 7.27 (m, 5H, 10-*m*1Ph & 5,15-*m*Ph); 7.13 (dd, 1H, ^3^*J*_HH_ = 8.3, 2.72 Hz, 10-*m*2Ph). HRMS (ESI): [M^+^] = 774.1274 (expt), 774.1273 (calcd for C_37_H_23_N_4_O_4_Re, major isotopomer).

#### Au[T*p*OHPC]

Yield 70.7 mg (70.2%). UV–vis (THF) λ_max_ (nm) and ε × 10^–4^ (M^−1^ cm^−1^): 420 (11.70), 559 (1.88), 580 (2.95). ^1^H NMR (400 MHz, 25 °C, THF-*d*_*8*_): *δ* 9.16 (m, 2H, *β*-H); 9.03 (d, 2H, ^3^*J*_HH_ = 4.8 Hz, *β*-H); 8.82 (d, 2H, ^3^*J*_HH_ = 4.4 Hz, *β*-H); 8.80 (d, 2H, ^3^*J*_HH_ = 4.8 Hz, *β*-H); 8.72(s, 2H, 5,15-*p*OHPh); 8.68 (s, 1H, 10-*p*OHPh); 8.07 (d, 4H, ^3^*J*_HH_ = 8.3 Hz, 5,15-*o*Ph); 7.95 (d, 2H, ^3^*J*_HH_ = 8.4 Hz, 10-*o*Ph); 7.21 (d, 4H, ^3^*J*_HH_ = 8.6 Hz, 5,15-*m*Ph); 7.16 (d, 2H, ^3^*J*_HH_ = 8.4 Hz, 10-*m*Ph). HRMS (ESI) [M^−^] = 767.1367 (expt), 767.1363 (calcd for C_37_H_23_N_4_O_3_Au, major isotopomer).

#### Re[T(3,5-OH)PC](O)

Yield 99.6 mg (90.1%). UV–vis (THF) λ_max_ (nm) and ε × 10^–4^ (M^−1^ cm^−1^): 440 (10.96), 556 (1.65), 587(2.24). ^1^H NMR (400 MHz, 25 °C, THF-*d*_*8*_): *δ* 9.64 (d, 2H, ^3^*J*_HH_ = 4.4 Hz, *β*-H); 9.44 (d, 2H, ^3^*J*_HH_ = 5.0 Hz, *β*-H); 9.42 (d, 2H, ^3^*J*_HH_ = 4.4 Hz, *β*-H); 9.24 (d, 2H, ^3^*J*_HH_ = 4.8 Hz, *β*-H); 8.67 (br s, 2H, 5,15(3,5-OHPh)_a_); 6.63 [s, 1H, 10(3,5-OH)_a_]; 8.47 [br s, 2H, 5,15(3,5-OHPh)_b_]; 8.42 [s, 1H, 10(3,5-OHPh)_b_]; 7.50 (br s, 2H, 5,15-*o*1Ph); 7.44 (s, 1H, 10-*o*1Ph); 6.92 (br s, 2H, 5,15-*o*2Ph); 6.77 (s, 1H, 10-*o*2Ph); 6.66 (m, 3H, 5,10,15-*p*Ph). HRMS (ESI): [M^−^] = 821.1046 (expt), 821.1053 (calcd for C_37_H_23_N_4_O_7_Re, major isotopomer).

#### Au[T(3,5*-*OH)PC]

Yield 60.4 mg (55.0%). UV–vis (THF) λ_max_ (nm) and ε × 10^–4^ (M^−1^ cm^−1^): 419(9.65), 576(2.64). ^1^H NMR (400 MHz, 25 °C, THF-*d*_*8*_): *δ* 9.22 (d, 2H, ^3^*J*_HH_ = 4.4 Hz, *β*-H); 9.19 (d, 2H, ^3^*J*_HH_ = 4.9 Hz, *β*-H); 8.98 (d, 2H, ^3^*J*_HH_ = 4.5 Hz, *β*-H); 8.94 (d, 2H, ^3^*J*_HH_ = 5.0 Hz, *β*-H); 8.50(s, 4H, 5,15(3,5-OHPh); 8.47 (s, 2H, 10(3,5-OHPh); 7.18 (d, 4H, ^4^*J*_HH_ = 2.2 Hz, 5,15-*o*Ph); 7.09 (d, 2H, ^4^*J*_HH_ = 2.2 Hz, 10-*o*Ph); 6.61 (m, 3H, ^3^*J*_HH_ = 8.6 Hz, 5,10,15-*p*Ph). HRMS (ESI): [M^−^] = 815.1206 (expt), 815.1210 (calcd for C_37_H_23_N_4_O_6_Au, major isotopomer).

#### Re[T*p*OFtPC](O)

Yield 53.8 mg (96.1%). UV–vis ((CH_2_Cl_2_/C_6_F_6_) λ_max_ (nm) and ε × 10^–4^ (M^−1^ cm^−1^): 442(8.78), 556(1.14), 590(1.64). ^1^H NMR (400 MHz, 25 °C, CDCl_3_): *δ* 9.58 (d, 2H, ^3^*J*_HH_ = 4.4 Hz, *β*-H); 9.31 (d, 4H, ^3^*J*_HH_ = 5 Hz, *β*-H); 9.11 (d, 2H, ^3^*J*_HH_ = 5 Hz, *β*-H); 8.50 (br s, 2H, 5,15-*o*1Ph); 8.43 (d, 1H, 10-*o*1Ph); 8.00 (br s, 2H, 5,15-*o*2Ph); 7.82 (d, 1H, 10-*o*2Ph); 7.47–7.29 (m, 5H, 10-*m*1Ph & 5,15-*m*Ph); 7.24 (d, 1H, ^3^*J*_HH_ = 8.4 Hz, 10-*m*2Ph); 4.35 (m, 6H, 5,10,15–OCH_2_–); 2.59 (m, 6H, 5,10,15-CH_2_-CF_2_-); 2.32 (m, 6H, 5,10,15–CH_2_–CH_2_O–); ^19^F NMR (C_6_F_6_): *δ* − 83.81, m 9F, CF_3_-); − 117.29, m, 6F, –CF_2_–; − 124.68, m, 6F, –CF_2_–; − 124.92, m 12F, –CF_2_–; − 125.71, m 6F, –CF_2_–; − 126.38, m 6F, –CF_2_–; − 129.22, m, 6F, –CF_2_–_._ HRMS (ESI): [M^+^] = 2154.1637 (expt), 2154.1636 (calcd for C_70_H_38_F_51_N_4_O_4_Re, major isotopomer).

#### Re[T(3,5-OFt)PC](O)

Yield 88.1 mg (94.6%). UV–vis (CH_2_Cl_2_/C_6_F_6_) λ_max_ (nm) and ε × 10^–4^ (M^−1^ cm^−1^): 440(9.17), 555(1.39), 585(1.89). ^1^H NMR (400 MHz, 25 °C, CDCl_3_): *δ* 9.59 (d, 2H, ^3^*J*_HH_ = 4.5 Hz, *β*-H); 9.38 (d, 4H, ^3^*J*_HH_ = 4.6 Hz, *β*-H); 9.18 (d, 2H, ^3^*J*_HH_ = 5.0 Hz, *β*-H); 7.77 (s, 2H, 5,15-*o*1Ph); 7.72 (s, 1H, 10-*o*1Ph); 7.25 (s, 2H, 5,15-*o*2Ph); 7.08 (s, 1H, 10-*o*2Ph); 6.92 (s, 3H, 5,10,15-*p*Ph) 4.39–4.09 (m, 12H, 5,10,15–OCH_2_–); 2.55–2.08 (m, 24H, 5,10,15–CH_2_–CH_2_–CF_2_–); ^19^F NMR (C_6_F_6_): *δ* − 83.98, m 18F, CF_3_–); − 117.40, m, 12F, –CF_2_–; − 124.78, m, 12F, –CF_2_–; − 125.07, m 24F, –CF_2_–; − 125.85, m 12F, –CF_2_–; − 126.52, m 12F, –CF_2_–; − 129.26, m, 12F, –CF_2_–_._ MS (ESI): [M^+^] = 3582.14 (expt), 3582.18 (calcd for C_103_H_53_F_102_N_4_O_7_Re, major isotopomer). Elemental analysis found C 34.58, H 1.47, N 1.52; calcd C 34.53, H 1.49, N 1.56.

#### Au[T*p*OFtPC]

Yield 53.0 mg (95.0%). UV–vis (CH_2_Cl_2_/C_6_F_6_) λ_max_ (nm) and ε × 10^–4^ (M^−1^ cm^−1^): 420 (9.35), 560(1.42), 580(2.20). ^1^H NMR (400 MHz, 25 °C, CDCl_3_): *δ* 9.19 (d, 2H, ^3^*J*_HH_ = 4.4 Hz, *β*-H); 9.03 (d, 2H, ^3^*J*_HH_ = 4.9 Hz, *β*-H); 8.86 (d, 2H, ^3^*J*_HH_ = 4.4 Hz, *β*-H); 8.80 (d, 2H, ^3^*J*_HH_ = 4.8 Hz, *β*-H); 8.21 (d, 4H, 5,15-*o*Ph); 8.10 (d, 2H, 10-*o*Ph); 7.34 (d, 4H, ^3^*J*_HH_ = 8.7 Hz, 5,15-*m*Ph); 7.29 (d, 2H, ^3^*J*_HH_ = 8.7 Hz, 10-*m*Ph); 4.34 (m, 6H, 5,10,15–OCH_2_–); 2.50 (m, 6H, 5,10,15–CH_2_–CF_2_–); 2.30 (m, 6H, 5,10,15–CH_2_–CH_2_O–); ^19^F NMR (C_6_F_6_): *δ* − 83.87, m 9F, CF_3_–); − 117.31, m, 6F, –CF_2_–; − 124.72, m, 6F, –CF_2_–; − 124.98, m 12F, –CF_2_–; − 125.78, m 6F, –CF_2_–; − 126.37, m 6F, –CF_2_–; − 129.17, m, 6F, –CF_2_–_._ HRMS (ESI): [M^+^] = 2148.1795 (expt), 2148.1790 (calcd for C_70_H_38_F_51_N_4_O_3_Au, major isotopomer).

#### Au[T(3,5-OFt)PC]

Yield 86.5 mg (93.1%). UV–vis (CH_2_Cl_2_/C_6_F_6_) λ_max_ (nm) and ε × 10^–4^ (M^−1^ cm^−1^): 419(10.48), 562(2.13), 572(2.35). ^1^H NMR (400 MHz, 25 °C, CDCl_3_): *δ* 9.22 (d, 2H, ^3^*J*_HH_ = 4.4 Hz, *β*-H); 9.11 (d, 2H, ^3^*J*_HH_ = 4.9 Hz, *β*-H); 8.95 (d, 2H, ^3^*J*_HH_ = 4.4 Hz, *β*-H); 8.88 (d, 2H, ^3^*J*_HH_ = 5.0 Hz, *β*-H); 7.47 (d, 4H, ^4^*J*_HH_ = 2.4 Hz, 5,15-*o*Ph); 7.38 (d, 2H, ^4^*J*_HH_ = 2.3 Hz, 10-*o*Ph); 6.87 (m, 3H, 5,10,15-*p*Ph); 4.24 (m, 12H, 5,10,15-OCH_2_–); 2.38 (m, 12H, 5,10,15-CH_2_–CF_2_–); 2.20 (m, 12H, 5,10,15-CH_2_–CH_2_O–); ^19^F NMR (C_6_F_6_): *δ* − 83.98, m 18F, CF_3_–); − 117.56, m, 12F, –CF_2_–; − 124.83, m, 12F, –CF_2_–; − 125.08, m 24F, –CF_2_–; − 125.88, m 12F, –CF_2_–; − 126.54, m 12F, –CF_2_–; − 129.30, m, 12F, –CF_2_–. MS (ESI): [M^+^] = 3577.2 (expt), 3577.2 (calcd for C_103_H_53_F_102_N_4_O_6_Au, major isotopomer) (Supplementary Information S1).

## Supplementary Information

Supplementary Information.

## Data Availability

All data generated or analyzed in this study are included in this published article and its supplementary information.
