# Supplementary material for: Phenol- and resorcinol-appended metallocorroles and their derivatization with fluorous tags
Source: Sci Rep. 2022 Nov 10;12:19256. doi: 10.1038/s41598-022-23889-0 (PMC9649713; doi:10.1038/s41598-022-23889-0)
Supplement: Supplementary file 1 — Supplementary Information. [file 41598_2022_23889_MOESM1_ESM.pdf]

Supporting information

Phenol- and Resorcinol-Appended Metallocorroles and Their Derivatization with Fluorous Tags

Abraham B. Alemayehu and Abhik Ghosh*

Department of Chemistry, University of Tromsø, N-9037 Tromsø, Norway;

Contents

Electrospray ionization mass spectra (8 compounds)

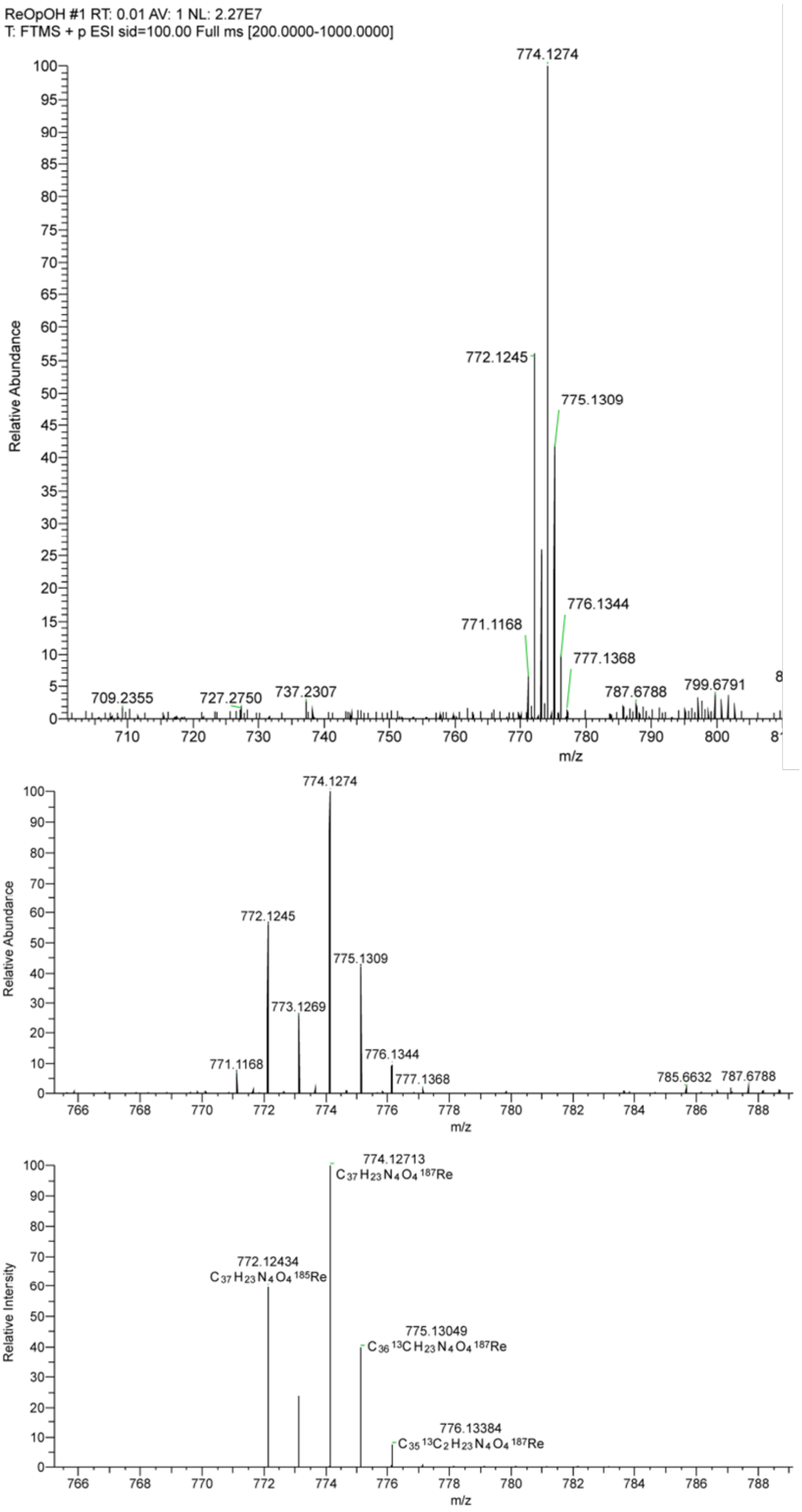

Figure S1. HRMS of Re[T*p*OHPC](O) in negative mode.

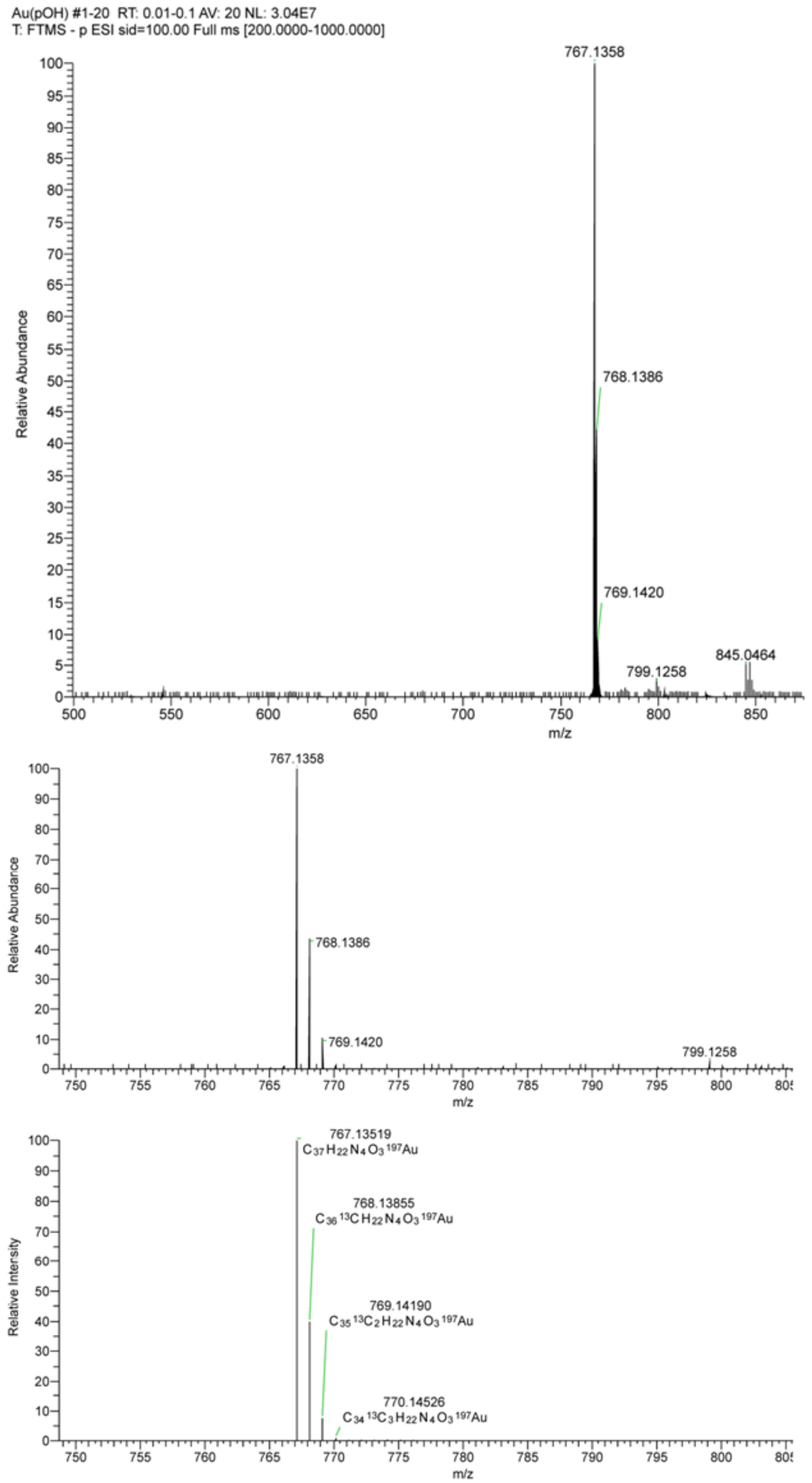

Figure S2. HRMS of Au[T*p*OHPC] in negative mode.

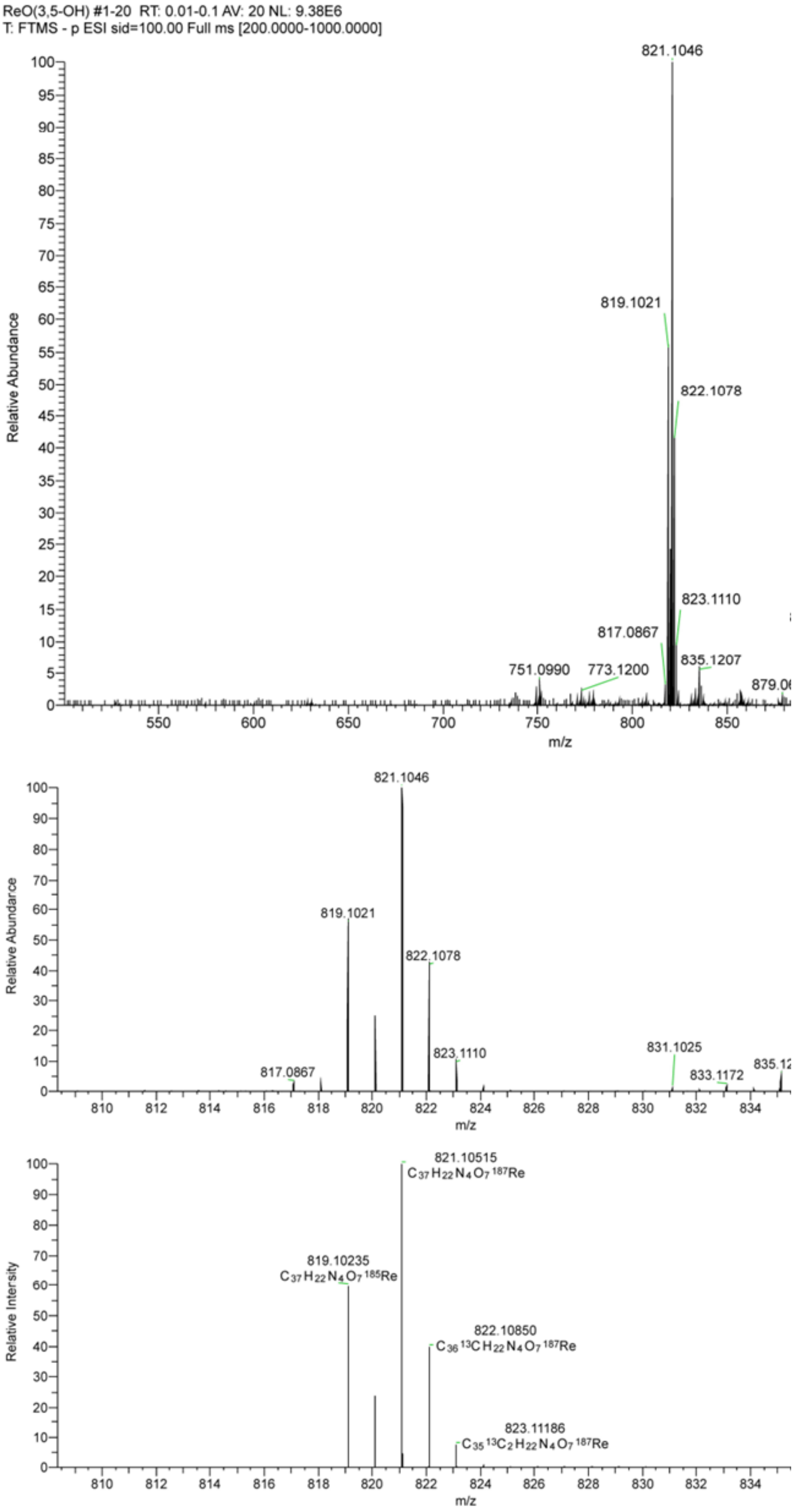

Figure S3. HRMS of Re[T(3,5-OH)PC](O) in negative mode.

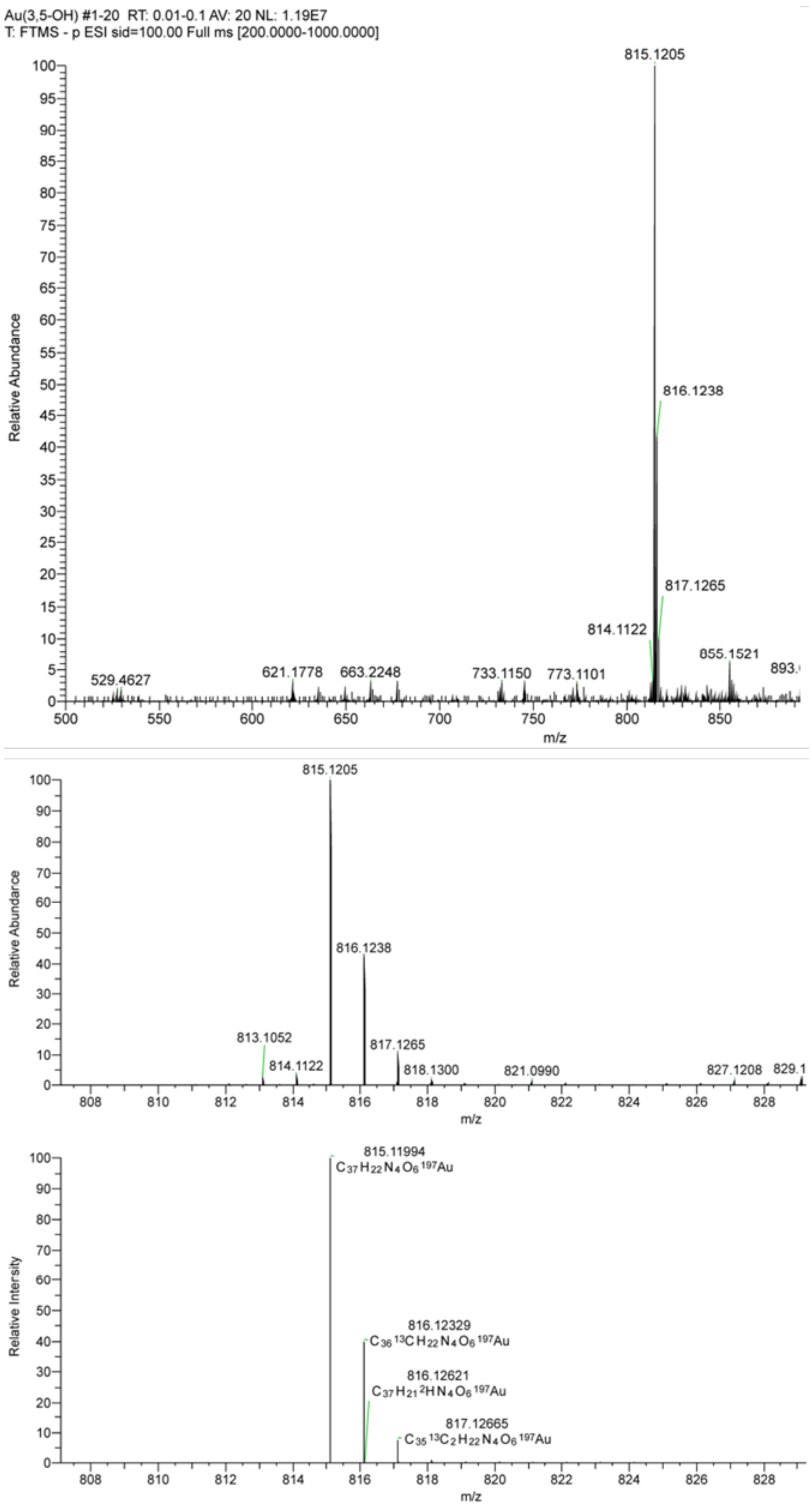

Figure S4. HRMS of Au[T(3,5-OH)PC] in negative mode.

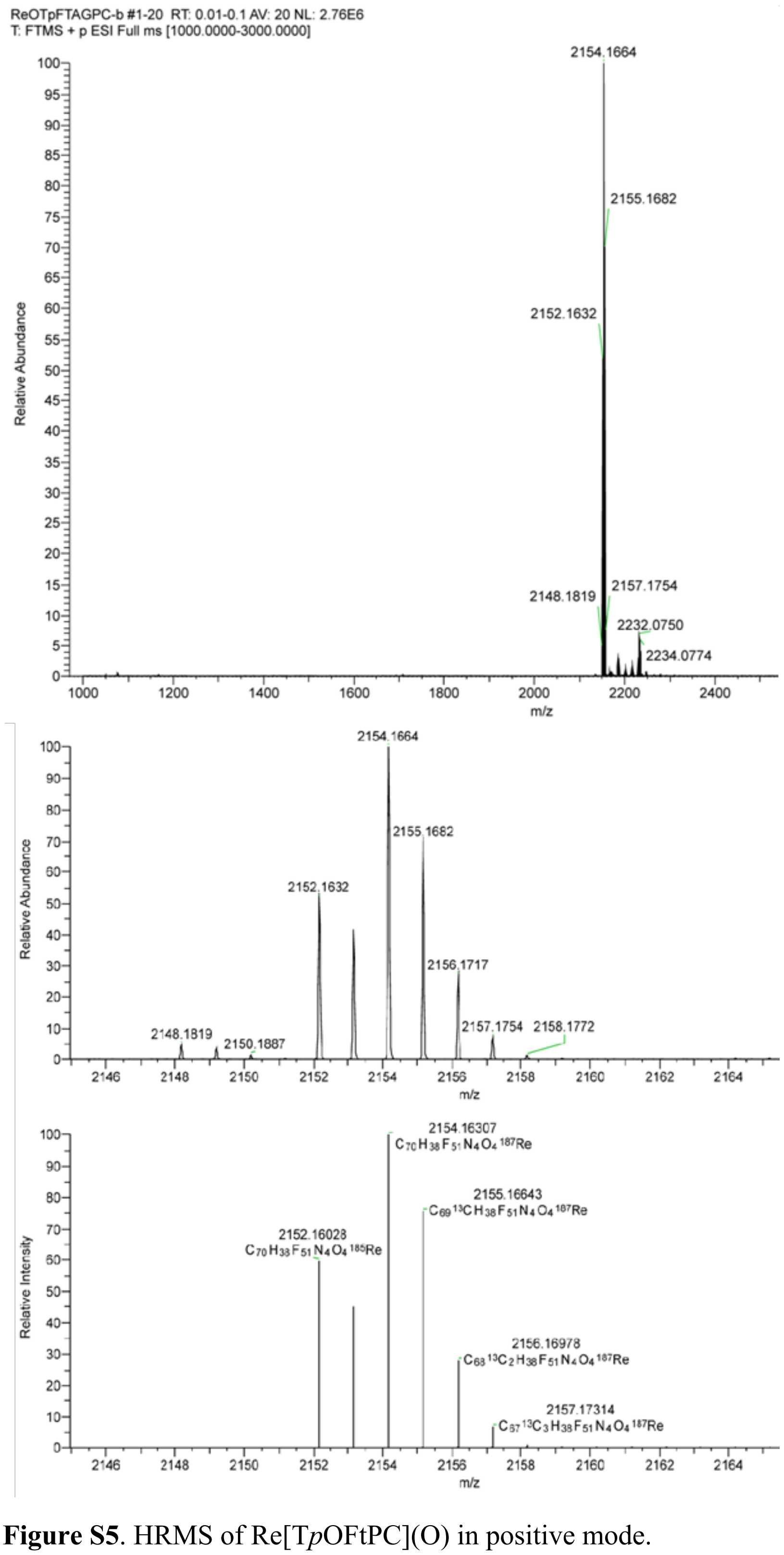

Figure S5. HRMS of Re[T*p*OFtPC](O) in positive mode.

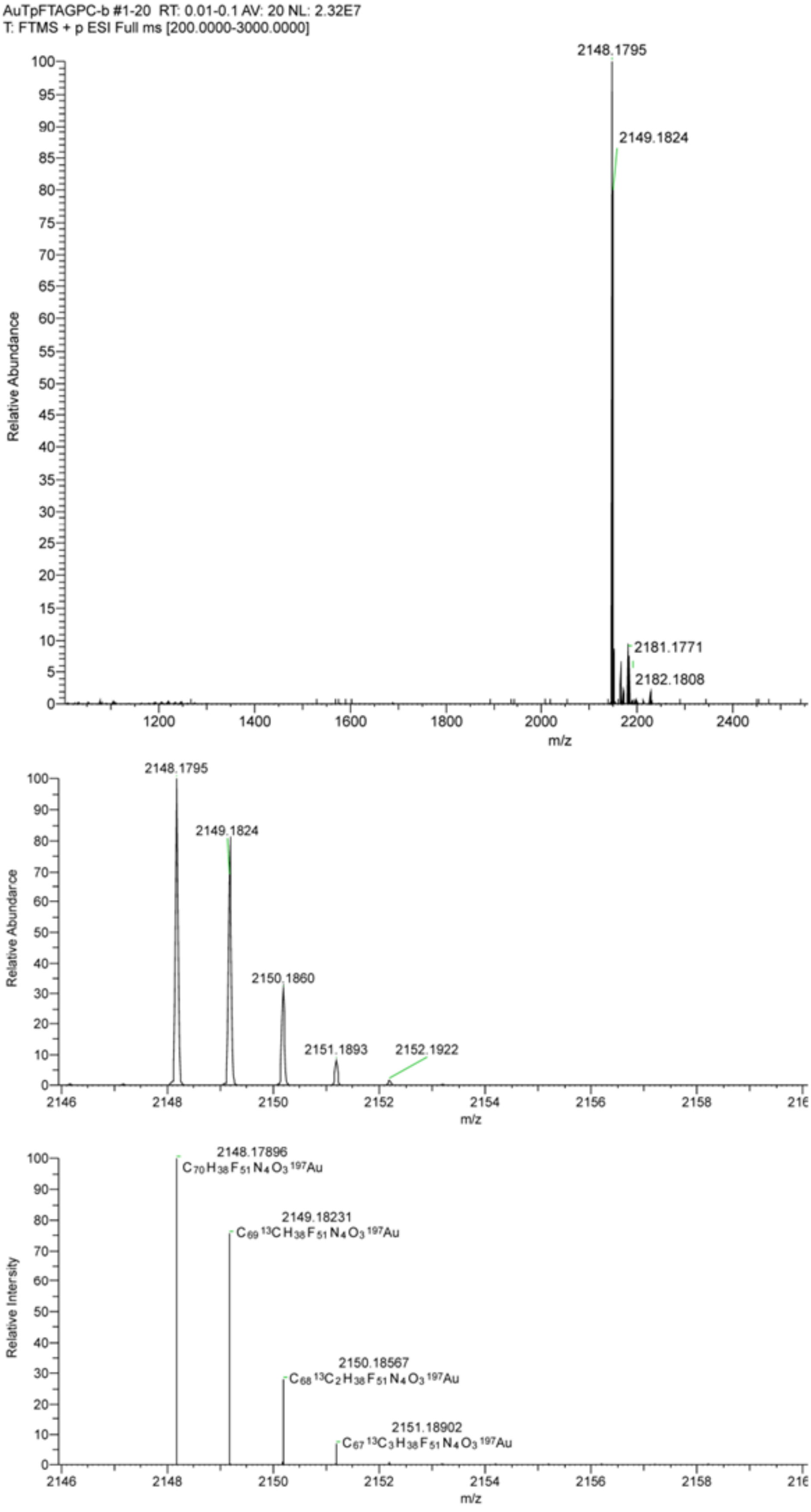

Figure S6. HRMS of Au[T*p*OFtPC] in positive mode.

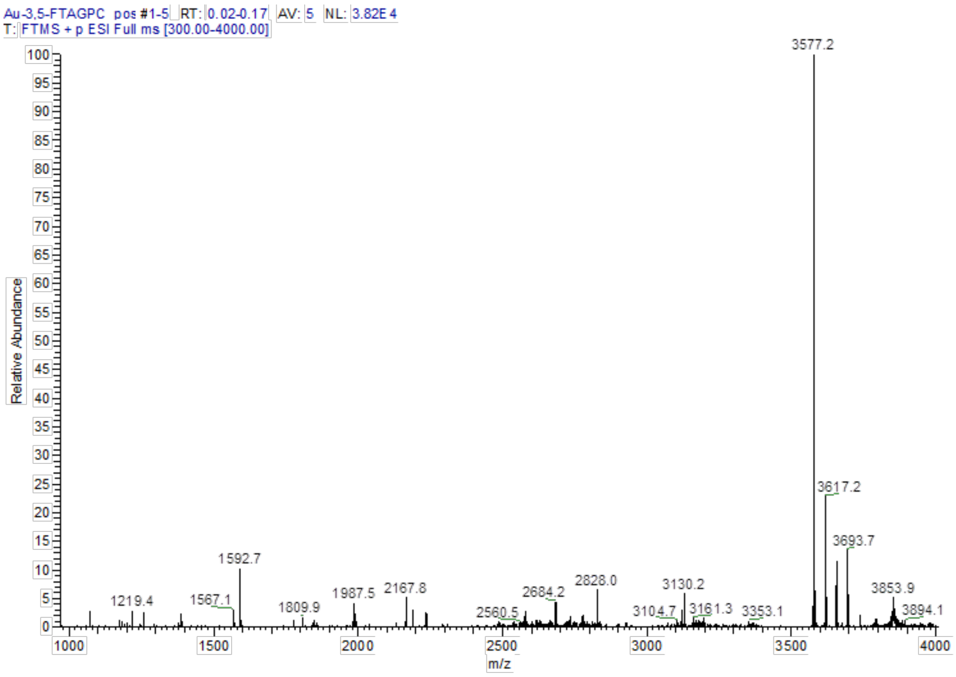

Figure S7. LRMS of Au[T(3,5-OFt)PC] in positive mode.

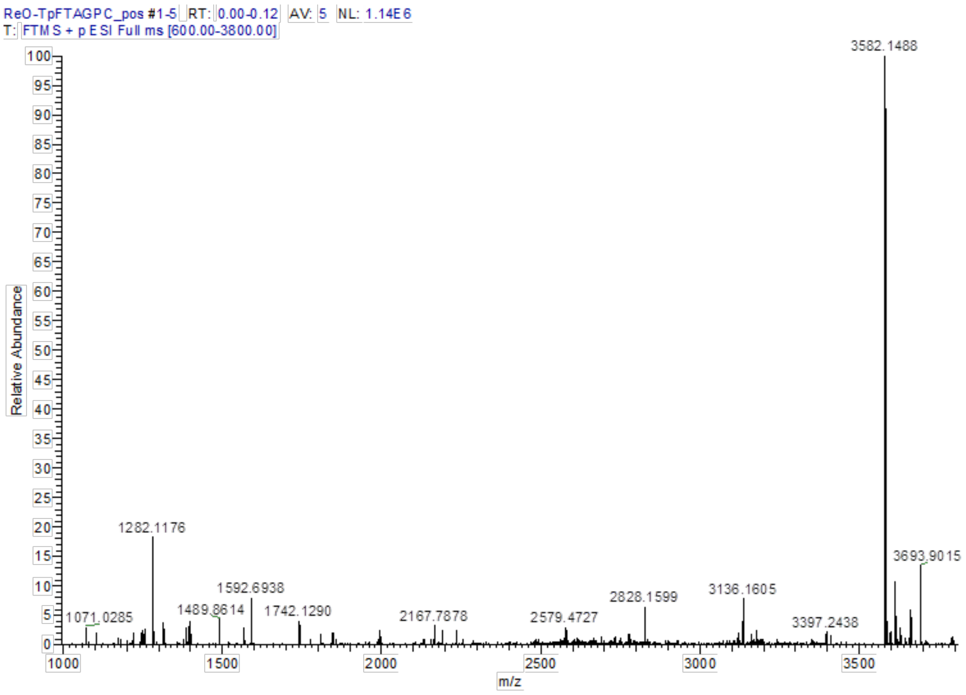

Figure S8. LRMS of Re[T{3,5-OFt)PC](O) in positive mode.
